# Women empowerment indices and utilization of health facilities during childbirth: evidence from the 2019 Sierra Leone demographic health survey

**DOI:** 10.1186/s12913-023-09122-2

**Published:** 2023-02-02

**Authors:** Quraish Sserwanja, David Mukunya, Milton W. Musaba, Linet M. Mutisya, Kassim Kamara, Shirin Ziaei

**Affiliations:** 1 Programmes Department, GOAL Global, Arkaweet Block 65 House No. 227, Khartoum, Sudan; 2 Department of Public Health, Busitema University, Mbale, Uganda; 3 Department of Research, Nikao Medical Center, Kampala, Uganda; 4 Department of Obstetrics and Gynaecology, Mbale Regional Referral and Teaching Hospital, Mbale, Uganda; 5 Department of Obstetrics and Gynaecology, Busitema University, Tororo, Uganda; 6 Maternal and Child Health Project, Swedish Organization for Global Health, Mayuge, Uganda; 7 7National Disease Surveillance Programme, Ministry of Health and Sanitation, Free Town, Sierra Leone; 8 Department of Women’s and Children’s Health, Uppsala University, Uppsala, Sweden

**Keywords:** Sierra Leone, DHS, Health facility utilization, Childbirth and women empowerment

## Abstract

**Background:**

Women empowerment is recognized as a potential enabling factor to the utilization of health facilities during childbirth. However, the association between women empowerment and utilization of health facilities is poorly studied, especially in counties with high maternal mortality. Therefore, we investigated the association between women empowerment indices and the utilization of health facilities during childbirth in Sierra Leone.

**Methods:**

We analyzed secondary data from the 2019 Sierra Leone Demographic and Health Survey (SLDHS). We included 5,997 married women who had given birth in the five years before the survey, and had been sampled for the women empowerment questionnaire. The study employed the gender roles framework developed by the Harvard Institute for International Development in the selection and classification of women empowerment indices, which include influencing, resource and decision-making factors. We conducted logistic regression analyses using SPSS version 25.0 complex samples package to determine the association between women empowerment indices and utilization of health facilities.

**Results:**

The overall prevalence of health facility utilization during childbirth was 84.1% (5,042/5,997): 95% CI: 83.6 to 85.4. Among the influencer domain variables, women from the southern (aOR = 2.25, 95% CI: 1.34–3.78), northern (aOR = 1.69,95% CI: 1.01–2.82) and eastern regions (aOR = 3.71, 95% CI: 2.03–6.77) had higher odds of health facility utilization compared to women in the western region, while women in polygamous marriages (aOR = 0.82, 95% CI: 0.69–0.98) had lower odds of utilizing health facilities compared to their counterparts in monogamous marriages. Furthermore, women who had their first birth when they were less than 18 years, had higher odds of utilizing health facilities (aOR = 1.22, 95% CI: 1.02–1.45) compared to those who were 18 years and above. Among the resource domain variables, women with post-primary education (aOR = 1.58, 95% CI: 1.21–2.06) had higher odds of utilizing health facilities compared to their counterparts with no education and women who belonged to the richest wealth quintile (aOR = 2.42, 95% CI: 1.31–4.46) had higher odds of utilizing health facilities compared to their counterparts belonging in the poorest quintile. None of the variables in the decision making domain was significantly associated with health facility utilization.

**Conclusion:**

These findings emphasize that, successful implementation of health facility utilization interventions should prioritize women empowerment with more pragmatic efforts. Policies and programme should aim at all women with more focus on those having lower education (primary and below), belonging to the poorest wealth quintile, give birth before reaching18 years and in polygamous marriages.

**Supplementary Information:**

The online version contains supplementary material available at 10.1186/s12913-023-09122-2.

## Background

Globally, approximately over 830 women die daily during pregnancy and in the postpartum period [1]. Most of these deaths are recorded in sub-Saharan Africa and are preventable [2]. The World Health Organization (WHO) recommends utilization of skilled care at health facilities during childbirth as one of the vital strategies for reducing maternal mortality and morbidity rates in low income settings [1, 2]. However, approximately 52% of childbirths in sub-Saharan Africa occur outside health facilities, conducted by mostly unskilled and under-equipped personnel [3, 4].

Women empowerment is a potential enabler of maternal healthcare seeking [5, 6], it also contributes to children’s health through improved nutrition and education [7, 8]. Low women empowerment status affects women’s health through the inability to participate in paid employment, experience of domestic violence, and limited access to maternal healthcare services through negative cultural beliefs and practices [9]. The gender roles framework developed by the Harvard Institute for International Development categorizes women’s empowerment variables into; influencing, resource and decision-making factors [10, 11]. Influencing factors include variables that are symbolic of gender norms and beliefs such as the gender division of labour, access, and control of resources [8] Resource factors include those that are related to human capital and access to resources while decision-making factors include women’s participation in the decision making process including access to and use of resources [10, 11].

There is growing recognition of the negative health outcomes of low women empowerment with gendered household power dynamics influencing access and utilization of maternal healthcare [12]. Using qualitative methodology, Cornish et al. explored the relationship between women’s economic empowerment and health decision-making in rural Sierra Leone and showed that although women’s economic empowerment was reported to ease marital tensions, men remained the main decision making figure in the household matters including healthcare seeking decision making [12]. Sierra leone has witnessed sluggish improvement in the women empowerment indices over the last decade with the percentage of currently married women who have employment having not changed at 85% but the percentage of women who receive cash from employment has doubled from 23% in 2008 to 45% in 2019 [13]. However, women are more likely than men to be unpaid (54% versus 27%), their decision making participation has declined from 73% in 2013 to 68% in 2019 while land ownership declined marginally from 36 to 32% [13].

Sierra Leone currently has one of the highest global maternal mortality ratio (MMR) standing at 717 deaths per 100,000 live births, while the neonatal mortality ratio (NMR) is 31 deaths per 1,000 live births respectively [13, 14]. Hence very far from achieving the Sustainable Development Goal (SDG) 3.1 of reducing global maternal mortality to less than 70 per 100,000 live births by 2030 [6, 15, 16]. Access to skilled birth attendance in a safe health facility environment for childbirth has been noted as one of the ways to help reduce maternal mortality [4]. Despite government efforts such as free maternal services that have averagely increased utilization of maternal and child health services over the last decade and have narrowed the inequity in antenatal (ANC) contacts and postnatal care (PNC) reviews, inequalities have been documented regarding institutional childbirths with less economically empowered women being the most affected [14]. However, there is no national study that has analysed the association of women empowerment on utilization of health facilities for childbirth. Therefore, it is of utmost importance to explore the influence of women's empowerment on health facility utilization during childbirth using the latest nationally representative dataset. Findings will enhance maternal health stakeholders’ understanding and also provide new perspectives for evidence based maternal health policy interventions. Therefore, in this study, we investigated the association between women empowerment indices and utilization of health facilities during childbirth among currently married women in Sierra Leone using the latest 2019 Sierra Leone Demographic and Health Survey (SLDHS). We hypothesize that empowered women are more likely to utilize health facilities during childbirth.

## Methods

### Study sampling and participants

Secondary data of the 2019 SLDHS, a nationally representative survey conducted from May 2019 to August 2019, was used in this cross sectional study [13]. Data about variables used in this study were collected using household and women’s questionnaires. A two-stage cluster sampling technique was used for the SLDHS with the census enumeration areas being the primary sampling units and households the secondary sampling units [13]. The enumeration areas were selected from the 2015 population and housing census sample frame [13]. Sampling weights were calculated based on sampling probabilities separately for each sampling stage and each cluster. A representative sample of 13,872 households was selected [13]. Tablets were used for household listing and computer programming was used to ensure random selection of households. The survey interviewers interviewed only the pre-selected households and to avoid bias, no replacements and no changes of the pre-selected households were allowed in the implementing stages. Before the main training to survey data collectors, pretest training was designed to prepare the trainers as well as to ensure that they were well versed with the SLDHS questionnaires and procedures and able to test the questionnaires in the different languages. Furthermore, majority of the data collectors had previous experience carrying out SLDHS surveys.

Women of reproductive age who were either permanent residents or had spent a night as a visitors in the sampled households were eligible to be interviewed. The men’s survey was conducted in one-half of the sample households and in this men subsample (half the sample households), one eligible woman in each household was randomly selected to be asked additional questions about domestic violence and other women empowerment indices [13]. A detailed description of the sampling process is available in the 2019 SLDHS report [13]. This secondary analysis included married women aged 15 to 49 years who had a live birth within five years preceding the survey (for those with more than one birth, the most recent birth was considered) and had been sampled for the women empowerment sub-questionnaire. Out of the total weighted sample of 15,574 women in the data set, 7,326 had given birth within five years preceding the survey**.** Of the 7,326 women, 5,997 women were married and had been sampled for the women empowerment sub-questionnaire.

#### Variables

##### Dependent variable

Health facility utilization during childbirth was the dependent variable. Birth outside health facility was referred to as home delivery and was coded as zero (0) while health facility delivery was coded as one (1) [4].

#### Independent variables

##### Women’s empowerment variables

The study employed the gender roles framework developed by the Harvard Institute for International Development in the selection and classification of women empowerment variables [10, 11, 17]. The framework categorizes women’s empowerment variables into;influencing, resource and decision-making factors.

Influencing factors included those that affect gender norms and beliefs and this study included; age (15–19, 20–34 and 35–49 years), residence (rural and urban), region (western, southern, northwestern, north and eastern) [18], religion (Islam and Christianity and others), sex of household head (male and female), and ≥ 18 years) and exposure to mass media (exposure to any of television, radio, newspapers or internet was a yes and non-exposure as a no).

Resource factors included those that are related to human capital and economic resources and these included; working status (working, not working), ownership of land or house (no, jointly only, both alone and jointly, and alone only), wealth index (poorest, poorer, middle, richer and richest quintiles) and level of education both for the women and their husbands (no education, primary, secondary and tertiary). Less than 3% of women had tertiary education hence secondary and tertiary were combined to make post-primary.

Decision-making variables included women’s participation in the decision regarding accessing healthcare, major household purchases, visits to family and relatives and spending of husband’s earnings. To give more specific insight into women empowerment as regards the different aspects of household decision-making power, each decision making variable was included as a single variable [10, 19]. These were categorized as; partner or someone else alone, woman alone and woman and partner.

### Statistical analysis

Statistical analysis was performed using SPSS complex samples software (version 25.0), which accounted for the multi-stage cluster study design by incorporating the sample individual weight, strata for sampling errors/design, and cluster number in the analysis plan [18, 20–22]. Use of weighted data accounted for the unequal probability sampling in different strata hence enabling representativeness of the SLDHS results at the national and regional levels.

Descriptive statistics were carried out to understand the background characteristics of the respondents in frequencies and percentages. To observe the crude and net effect of the different domains of women empowerment on health facility utilization during childbirth, each empowerment domain was modelled separately and a full model was estimated using all the variables with bivariable level of significance set at *p*-value < 0.25 [10].

Four logistic regression models were estimated with model one having influencer variables, model two having resource variables, model three having decision-making variables and model four adjusted for confounders in addition to having all the variables in model 1, 2 and 3. Confounding factors known to be associated with health facility utilization from the literature and those found significant at a p-value less than 0.25 at bivariable logistic regression analysis such as (problems with distance to the nearest health facility (big problem and no problem), children ever born (less than 2, 2–4 and 5 and above), being visited by a field health worker (yes and no), number of household members (less than 7 and 7 and above) and antenatal care (ANC) attendance (less than 8 and 8 and above contacts) were included in the multivariable logistic regression models.The multivariable regression results were presented by the estimated adjusted odds ratios (aOR) with 95% confidence interval (CI) and statistical significance level was set at *p*-value < 0.05. We assessed all covariates for collinearity, using a cut off value of above 5 variance inflation factor. Sensitivity analysis was also done by looking at only first time mothers.

## Results

### Socio-demographic characteristics

A total of 5,997 women were included in this study, and the prevalence of health facility utilization during childbirth was (5,042/5,997), 84.1% (95% CI: 83.6–85.4) as shown in Table**1**. The majority of the women were aged 20 to 34 years (65.9%), resided in rural areas (65.5%), belonged to Islam (80.6%), were in monogamous marriages (71.7%), had no exposure to mass media (52.9%), were working (80.7%), owned no house or land (51.1% and 57.4% respectively) and had no education (58.4%). Most of the women were not participating in decision making regarding accessing healthcare (57.7%), major household purchases (54.9%), visiting family and friends (55.3%) and spending on partner’s earnings (62.8%).

**Table 1:** Socio-demographic characteristics of women as per the 2019 SLDH

Characteristics	*n* = 5997	%
**Influencing variables**		
**Age**		
35 to 49	1734	28.9
20 to 34	3954	65.9
15 to 19	309	5.2
**Residence**		
Rural	3925	65.5
Urban	2072	34.5
**Region**		
Western	1079	18.0
Southern	1204	20.1
Northwestern	1196	19.9
Northern	1252	20.9
Eastern	1266	21.1
**Religion**		
Islam	4846	80.8
Christianity and others	1151	19.2
**Sex household head**		
Male	4882	81.4
Female	1115	18.6
**Marriage type**		
Monogamy	4301	71.7
Polygamy	1696	28.3
**Exposure to mass media**		
No	3173	52.9
Yes	2824	47.1
Age at first birth		
Less than 18	2226	37.1
18 and above	3771	62.9
**Resource Variables**		
**Working status**		
Not working	1159	19.3
Working	4838	80.7
**Owning a house**		
No	3067	51.1
Jointly only	2164	36.1
Both alone and jointly	463	7.7
Alone only	303	5.1
**Owning land**		
No	3442	57.4
Jointly only	1873	31.2
Both alone and jointly	331	5.5
Alone only	352	5.9
**Education Level**		
No Education	3502	58.4
Primary Education	869	14.5
Post-primary Education	1625	27.1
**Husband Education**		
None	3423	57.1
Primary	462	7.7
Secondary	1645	27.4
Tertiary	468	7.8
**Wealth Index**		
Poorest	1407	23.5
Poorer	1336	22.3
Middle	1247	20.8
Richer	1060	17.7
Richest	947	15.8
**Decision making**		
**Accessing healthcare**		
Partner alone or someone else	3458	57.7
Woman alone	511	8.5
Woman and Partner/someone else	2028	33.8
**Major purchases**		
Partner alone or someone else	3294	54.9
Woman alone	678	11.3
Woman and Partner/someone else	2025	33.8
**Visiting family or relatives**		
Partner alone or someone else	3315	55.3
Woman alone	712	11.9
Woman and Partner/someone else	1970	32.8
**Spending husband’s earnings**^**a**^		
Partner alone or someone else	3691	62.8
Woman alone	453	7.7
Woman and Partner/someone else	1737	29.5
**Confounders**		
**Distance to health facility**		
Big problem	2914	48.6
Not big problem	3083	51.4
**Chidren everborn**		
5 and above	1240	20.7
2–4	3582	59.7
Less than 2	1175	19.6
**Visited by field workers**		
No	4139	69.0
Yes	1858	31.0
**ANC attendance**		
Less than 8 contacts	4680	78.0
8 and above contacts	1317	22.0
Number of household members		
Less than 7	3400	56.7
7 and above	2597	43.3
**Outcome**		
**Health facility utilization**		
Yes	5042	84.1
No	955	15.9

^a^missing 117

### Influence of women’s empowerment indices on the utilization of health facilities during childbirth

Considering indices’ specific models, in the influencers’s model, belonging to northwestern region and to a polygamous household was associated with less odds while mass media exposure, belonging to the southern and eastern regions, urban areas and younger age groups were associated with higher odds of health facility utilization while among resources’model, owning a house alone, primary and post primary education levels, belonging to richer and richest wealth quintiles were associated with her odds of health facility utilization. In the decision making model, accessing healthcare decision making was the only significant variable with women who were making decisions alone having less odds of health facility utilization.

In the final adjusted model that included all factors, among the influencer domain variables, women from the southern (aOR = 2.25, 95% CI: 1.34–3.78), northern (aOR = 1.69,95% CI: 1.01–2.82) and eastern regions (aOR = 3.71, 95% CI: 2.03–6.77) had higher odds of of health facility utilization compared to the women in the western region while women in polygamous marriages (aOR = 0.82, 95% CI: 0.69–0.98) had less odds of utilizing health facilities compared to their counterparts in monogamous marriages. Furthermore, women who had their first birth when they were less than 18 years, had higher odds of utilizing health facilities (aOR = 1.22, 95% CI: 1.02–1.45) compared to those who were 18 years and above. Among the resource domain variables, women with post-primary level of education (aOR = 1.58, 95% CI: 1.21–2.06) and belonged to the richest wealth quintile (aOR = 2.42, 95% CI: 1.31–4.46) had significantly higher odds of utilizing health facilities compared to their counterparts with no education and those belonging in the poorest quintile respectively. None of the variables in the decision making domain was significantly associated with health facility utilization as shown in Table 2 and Supplementary file 1.

**Table 2:** Influence of women’s empowerment indices on the utilization of health facilities during childbirth in Sierra Leone

Characteristics	*N* = 5997	*N* = 5997	*N* = 5997	*N* = 5997	*N* = 5997
**Influencer variables**	**Bivariable cOR (95% CI)**	**Influencer domain** **aOR (95%CI)**	**Resource domain** **aOR (95%CI)**	**Decision domain** **aOR (95%CI)**	**Full model** **aOR (95%CI)**
**Age**					
35 to 49	1				1
20 to 34	**1.39 (1.17–1.65)**^**†**^	1.26 (1.05–1.52)*			1.10 (0.88–1.36)
15 to 19	**1.55 (1.04–2.30)**^**†**^	1.50 (0.98–2.31)			1.23 (0.75–2.01)
**Residence**					
Rural	1	1			1
Urban	**2.12 (1.60–2.81)**^**‡**^	**1.95 (1.41–2.68)**^**‡**^			1.18 (0.80–1.75)
**Region**					
Western	1	1			1
Southern	0.75 (0.48–1.15)	1.64 (1.01–2.66)*			**2.25 (1.34–3.78)**^**†**^
Northwestern	**0.27 (0.18–0.40)**^**‡**^	0.56 (0.36–0.88)*			0.71 (0.43–1.16)
Northern	0.66 (0.43–1.01)	1.34 (0.83–2.16)			1.69 (1.01–2.82)*
Eastern	1.42 (0.81–2.51)	**2.86 (1.60–5.14)**^**‡**^			**3.71 (2.03–6.77)**^**‡**^
**Religion**					
Islam	1	1			1
Christianity and others	**1.46 (1.11–1.92)**^**†**^	1.00 (0.75–1.34)			0.91 (0.68–1.22)
**Sex household head**^**a**^					
Male	1	-			-
Female	0.89 (0.72–1.11)				
**Marriage type**					
Monogamy	1	1			1
Polygamy	**0.61 (0.51–0.72)**^**‡**^	**0.76 (0.64–0.91)**^**†**^			0.82 (0.69–0.98)*
**Mass media exposure**					
No	1	1			1
Yes	**1.66 (1.33–2.07)**^**‡**^	**1.44 (1.15–1.80)**^**†**^			1.18 (0.95–1.48)
Age at first birth					
18 and above	**1**	**1**			**1**
Less than 18	1.2 (1.03–1.40)*	**1.16 (0.98–1.36)**			1.22 (1.02–1.45)*
**Resource Variables**					
**Working status**					
Not working	1		1		1
Working	0.77 (0.60–0.99)*		1.04 (0.81–1.33)		1.00 (0.78–1.29)
**Owning a house**					
No	1		1		1
Jointly only	0.88 (0.73–1.06)		1.12 (0.88–1.42)		1.14 (0.89–1.46)
Both alone and jointly	**0.62 (0.45–0.86)**^**†**^		0.95 (0.63–1.42)		0.85 (0.57–1.26)
Alone only	1.39 (0.89–2.17)		1.59 (1.01–2.52)*		1.29 (0.79–2.09)
**Owning land**					
No	1		1		1
Jointly only	0.78 (0.64–0.96)*		1.00 (0.77–1.30)		1.01 (0.77–1.33)
Both alone and jointly	**0.49 (0.34–0.71)**^**‡**^		0.73 (0.47–1.12)		1.01 (0.63–1.60)
Alone only	1.06 (0.70–1.59)		1.19 (0.77–1.83)		1.00 (0.62–1.60)
**Education Level**					
No Education	1		1		1
Primary Education	**1.48 (1.15–1.91)**^**‡**^		1.37 (1.07–1.76)*		1.26 (0.99–1.61)
Post-primary Education	**2.42 (1.89–3.09)**^**‡**^		**1.79 (1.41–2.28)**^**‡**^		**1.58 (1.21–2.06)**^**†**^
**Husband Education**					
None	1		1		1
Primary	**1.58 (1.15–2.18)**^**†**^		1.33 (0.96–1.83)		1.22 (0.87–1.71)
Secondary	**1.65 (1.28–2.13)**^**‡**^		1.14 (0.89–1.46)		1.06 (0.82–1.37)
Tertiary	**2.60 (1.71–3.94)**^**‡**^		1.33 (0.86–2.06)		1.24 (0.79–1.94)
**Wealth Index**					
Poorest	1		1		1
Poorer	1.11 (0.90–1.36)		1.06 (0.86–1.32)		1.26 (1.01–1.57)*
Middle	1.29 (0.99–1.68)		1.20 (0.92–1.56)		1.33 (1.02–1.74)*
Richer	**1.73 (1.28–2.33)**^**‡**^		1.45 (1.06–1.20)*		1.34 (0.90–1.98)
Richest	**3.37 (2.14–5.33)**^**‡**^		**2.53 (1.56–4.09)**^**‡**^		**2.42 (1.31–4.46)**^**†**^
**Decision making**					
**Accessing healthcare**					
Partner alone	1			1	1
Woman alone	0.79 (0.57–1.09)			0.60 (0.39–0.92)*	0.81 (0.51–1.27)
Woman and Partner	1.13 (0.92–1.38)			0.96 (0.69–1.33)	0.98 (0.70–1.37)
**Major purchases**					
Partner alone	1			1	1
Woman alone	0.99 (0.76–1.30)			1.10 (0.79–1.53)	1.27 (0.91–1.79)
Woman and Partner	1.18 (0.96–1.46)			1.16 (0.82–1.65)	1.34 (0.99–1.82)
**Visiting**					
Partner alone	1			1	1
Woman alone	1.21 (0.88–1.66)			1.45 (0.96–2.21)	1.01 (0.66–1.53)
Woman and Partner	1.14 (0.93–1.40)			1.07 (0.77–1.49)	0.86 (0.63–1.18)
**Spending husband’s earnings**					
Partner alone	1			-	
Woman alone	0.87 (0.64–1.18)				
Woman and Partner	1.09 (0.88–1.36)				

a *p*-value above 0.25 at bivariable level, *p-value < 0.05, † *p*-value < 0.01 ‡ *p*-value < 0.001. Full model included confounders; parity, distance to health facility, ANC attendance and visit by field health worker.

In the sensitivity analysis that included only women giving birth for the first time, the average age was 22 years and prevalence of health facility utilization was 87.5% (95% CI: 85.6–89.8) with only one influencing domain variable (region; women in Eastern region (aOR = 3.63, 95% CI: 1.09–12.09), and one resource domain variable (education; with women having post-primary level of education (aOR = 2.18, 95% CI: 1.15–4.15) being significantly associated with higher odds of utilizing health facilities for childbirth.

## Discussion

This study examined the association between women’s empowerment indices and the utilization of health facilities during childbirth in Sierra Leone with indices being measured in the three.

domains of influencer, resource and decision-making factors. The prevalence of health facility utilization during childbirth was 84.1% (95% CI: 83.6–85.4). This is higher than similar national DHS studies in Kenya [23], Uganda [4], and Ghana [24]. This could be attributed to the fact that most of these studies used earlier DHS data sets and health facility utilization has been increasing over time. Furthermore, the differences in access to healthcare facilities, health-seeking behavior, and economic development among these countries could also partly explain the observed differences.

Women empowerment indices from the influencer and resource domains were associated with utilization of health facilities during child birth. Among the influencer factors, women from the Southern and Eastern regions had higher odds of utilizing health facilities compared to those from the Western region while women in polygamous marriages had less odds of utilizing health facilities compared to their counterparts in monogamous marriages. The region as a determinant of maternal health facility utilization during childbirth has been shown in similar studies in Ghana, Uganda and India [4, 6, 24]. Post-conflict in Sierra Leone, the health system has been described as fragile [25]. The western region is the most developed with a high concentration of skilled health workers compared to other regions [26, 27]. Rapid urbanisation, has also resulted into a high number of the urban poor seeking for low paying jobs,which has led to inequitable access and affordability of basic services [28–30]. This inequitable access to basic services has a great effect on the urban poor [13]. In addition, the western region being seen as better off than the other regions in terms of service delivery has led to health non-governmental organizations focusing their support in other regions which factor could also partly explain this finding.

Women who were in polygamous marriages had less odds of utilizing health facilities compared to their counterparts in monogamous marriages. Similarly, polygamy has been shown by Stephen et al. in Nigeria [31] and Lowe et al. [32] in The Gambia to have a negative impact on maternal healthcare utilization. Dilution and scarcity of resources in polygamous families are common mechanisms that are used to explain the decreased likelihood of health facility utilization among women in polygamous relationships [12, 31, 33]. This has been debated against in some literature with some reporting that some polygamous men tend to be wealthy [34]. However, even if polygynous men tend to be richer than their monogamous counterparts, the bigger family size in polygamous families dilutes the per capita resources which negatively affects household savings that can be used to cater for the direct and indirect costs associated with accessing care [31, 34]. Furthermore, the gender asymmetry that is documented in polygamous families reflects gender inequalities and unbalanced power differentials within families. Such inbalance might contribute to low healthcare utilization through limited paternal involvement in discussing reproductive decisions and reducing household workload to avail women enough time to seek care and investment in their health [32, 34, 35]. Women who had their first birth when they were less than 18 years, had higher odds of utilizing health facilities compared to those who were 18 years and above. This could be attributed to the fact that girls below 18 years tend to have their first childbirths out of health facilities with unskilled birth attendants [36, 37], which risks negative childbirth experiences hence their increased likelihood of using health facilities for their subsequent childbirths.

Among the resource variables, women with post-primary levels of education and those belonging to the richest wealth quintile had higher odds of utilizing health facilities compared to their counterparts with no education and those belonging in the poorest quintile respectively. This result is consistent with those of previous studies suggesting that higher levels of education are associated with health facility utilization of maternal healthcare services [4, 24, 38]. Higher levels of education have been shown to empower women through better employment opportunities, women being more open to receiving new health related information positively and more awareness of women about available health resources, and more knowledge about health behaviors and the healthcare system which leads to better maternal literacy and health seeking behavior [4, 9, 38]. Furthermore, these women have greater chances to effectively cope with or challenge issues presented by negative socio-cultural beliefs and norms that might affect access to healthcare [39]. Therefore, maternal healthcare stakeholders in Sierra Leone need to promote girl child education to ensure that a high proportion of girls can at least finish secondary level of education.

Similar to previous studies, wealth index has been positively associated with maternal healthcare service utilization [4, 9, 15, 40, 41]. Besides easier access to resources and basic services including healthcare, women belonging to the highest wealth quintiles have been shown to have enhanced social status hence high decision-making powers, more involvement in large household purchases, and are more likely to have a final say in their own health care [42–44]. Since Sierra Leone provides free maternal healthcare services in public facilities [12], these results further highlight the high influence of indirect financial costs such as transport fees in accessing and utilizing health facilities for less economically empowered women. These costs can deter women from accessing quality maternal care throughout pregnancy creating a threat to maternal mortality and morbidity reduction interventions.

Suprisingly, women empowerment indices from the the decision making domain were not associated with health facility utilization. At bivariable analysis; women who made decisions together with their partners had higher odds of health facility births. However, this association was absent at multivariable analysis which suggests that factors such as maternal education might have confounded that association.This suprising result could have resulted from the changing landscape of health facility births in Sierra Leone. At a health facility birth rate of 84%, it is likely that majority of the people know the benefits of health facility birth. As such, the barriers to health facility birth are more of resource related and not “decision” related. This is supported by the fact that decision making in purchases (which is related to resources) is associated with health facility birth. Also, decision making is a complex process that is embedded in multiple cultural constructs. In certain cultures (ref); sole maternal decision making might be beneficial, however, in other setting; sole maternal decision making might represent paternal apathy.

### Strengths and limitations

This is the foremost nationwide analysis that explores the association of women empowerment with utilization of health facilities during childbirth. Therefore, it can be used as a yardstick and motivation for further studies on the related subject matter. Secondly, we used the most current nationally representative data hence the findings are generalizable to all married women in Sierra Leone. However, the use of cross-sectional data only enables associations to be established but not causal relationships. Additionally, it needs to be acknowledged that women empowerment is a complex phenomenon and spreads across several dimensions including social, economic, and political at micro, meso and macro level [45]. While Harvard analytical framework is a useful tool for collecting data at the micro level, it does not reflect deteminants of women empowerment at meso and macro level. Further studies evaluating the association between indicators of women empowerment and their health care utilization during childbirth at all these levels are warranted. Lastly, the possibility of recall bias due to self-reported answers and lack of important variables such as perceived quality of received ANC,women’s perception of quality of education, starting up and owning businesses and other empowerment indicators not available in DHS could be a limitation in this study.

## Conclusion

This study has highlighted that the utilization of health facilities during childbirth is high and above that of many countries in the region. Only two empowerment domains have shown to be associated with health facility utilization. Policies and programme should aim at all women with more focus on those in western Sierra Leone, those having lower education (primary and below), having had their first childbirth when below 18 years, belonging to lowest wealth quintile and in polygamous marriages. Maternal health stakeholders may need to invest in and prioritize programmes aimed at ensuring that more girls acquire education beyond primary level and also preventing teenage pregnancies. Strengthening maternal community based health services or construction of more health facilities could be helpful in ensuring easier access to skilled birth attendance by reducing effects of barriers such as indirect costs of transportation which may hinder women from utilizing health facilities. Qualitative studies are recommended to further explore the the links demonstrated by this quantitative analysis.

## Supplementary Information

**Additional file 1.** Influence of women’s empowerment indices on the utilization of health facilities during childbirth in Sierra Leone.

## Data Availability

The data set used is openly available upon permission from MEASURE DHS website (URL: https://www.dhsprogram.com/data/available-datasets.cfm). However, authors are not authorized to share this data set to the public but anyone interested in the data set can seek it with written permission from MEASURE DHS.

## Abbreviations

EA: Enumeration area
AOR: Adjusted odds ratio
CI: Confidence interval
COR: Crude odds ratio
DHS: Demographic health survey
SLDHS: Sierra leone demographic health survey
OR: Odds ratio
SD: Standard deviation
WHO: World health organization
ANC: Antenatal care
PNC: Postnatal care
SBA: Skilled birth attendance
CoC: Continuum of care
SPSS: Statistical package for social science
