# Supplementary material for: Women empowerment indices and utilization of health facilities during childbirth: evidence from the 2019 Sierra Leone demographic health survey
Source: BMC Health Serv Res. 2023 Feb 2;23:109. doi: 10.1186/s12913-023-09122-2 (PMC9893537; doi:10.1186/s12913-023-09122-2)
Supplement: Supplementary file 1 — Additional file 1. Influence of women’s empowerment indices on the utilization of health facilities during childbirth in Sierra Leone. [file 12913_2023_9122_MOESM1_ESM.docx]

**Influence of women’s empowerment indices on the utilization of health facilities during childbirth in Sierra Leone**

| **Characteristics** | **N=5997** | **N=5997** |
| --- | --- | --- |
| **Influencer variables** | **Bivariable**  **cOR (95% CI)** | **Full model**  **aOR (95%CI)** |
| **Age** |  |  |
| 35 to 49 | 1 | 1 |
| 20 to 34 | **1.39 (1.17-1.65)^†^** | 1.10 (0.88-1.36) |
| 15 to 19 | **1.55 (1.04-2.30)^†^** | 1.23 (0.75-2.01) |
| **Residence** |  |  |
| Rural | 1 | 1 |
| Urban | **2.12 (1.60-2.81)^‡^** | 1.18 (0.80-1.75) |
| **Region** |  |  |
| Western | 1 | 1 |
| Southern | 0.75 (0.48-1.15) | **2.25 (1.34-3.78)^†^** |
| Northwestern | **0.27 (0.18-0.40)^‡^** | 0.71 (0.43-1.16) |
| Northern | 0.66 (0.43-1.01) | **1.69 (1.01-2.82)*** |
| Eastern | 1.42 (0.81-2.51) | **3.71 (2.03-6.77)^‡^** |
| **Religion** |  |  |
| Islam | 1 | 1 |
| Christianity and others | **1.46 (1.11-1.92)^†^** | 0.91 (0.68-1.22) |
| **Sex household head^a^** |  |  |
| Male | 1 | - |
| Female | 0.89 (0.72-1.11) |  |
| **Marriage type** |  |  |
| Monogamy | 1 | 1 |
| Polygamy | **0.61 (0.51-0.72)^‡^** | **0.82 (0.69-0.98)*** |
| **Mass media exposure** |  |  |
| No | 1 | 1 |
| Yes | **1.66 (1.33-2.07)^‡^** | 1.18 (0.95-1.48) |
| Age at first birth |  |  |
| 18 and above | **1** | **1** |
| Less than 18 | **1.2 (1.03-1.40)*** | **1.22 (1.02-1.45)*** |
| **Resource Variables** |  |  |
| **Working status** |  |  |
| Not working | 1 | 1 |
| Working | **0.77 (0.60-0.99)*** | 1.00 (0.78-1.29) |
| **Owning a house** |  |  |
| No | 1 | 1 |
| Jointly only | 0.88 (0.73-1.06) | 1.14 (0.89-1.46) |
| Both alone and jointly | **0.62 (0.45-0.86)^†^** | 0.85 (0.57-1.26) |
| Alone only | 1.39 (0.89-2.17) | 1.29 (0.79-2.09) |
| **Owning land** |  |  |
| No | 1 | 1 |
| Jointly only | **0.78 (0.64-0.96)*** | 1.01 (0.77-1.33) |
| Both alone and jointly | **0.49 (0.34-0.71)^‡^** | 1.01 (0.63-1.60) |
| Alone only | 1.06 (0.70-1.59) | 1.00 (0.62-1.60) |
| **Education Level** |  |  |
| No Education | 1 | 1 |
| Primary Education | **1.48 (1.15-1.91)^‡^** | 1.26 (0.99-1.61) |
| Post-primary Education | **2.42 (1.89-3.09)^‡^** | **1.58 (1.21-2.06)^†^** |
| **Husband Education** |  |  |
| None | 1 | 1 |
| Primary | **1.58 (1.15-2.18)^†^** | 1.22 (0.87-1.71) |
| Secondary | **1.65 (1.28-2.13)^‡^** | 1.06 (0.82-1.37) |
| Tertiary | **2.60 (1.71-3.94)^‡^** | 1.24 (0.79-1.94) |
| **Wealth Index** |  |  |
| Poorest | 1 | 1 |
| Poorer | 1.11 (0.90-1.36) | **1.26 (1.01-1.57)*** |
| Middle | 1.29 (0.99-1.68) | **1.33 (1.02-1.74)*** |
| Richer | **1.73 (1.28-2.33)^‡^** | 1.34 (0.90-1.98) |
| Richest | **3.37 (2.14-5.33)^‡^** | **2.42 (1.31-4.46)^†^** |
| **Decision making** |  |  |
| **Accessing healthcare** |  |  |
| Partner alone | 1 | 1 |
| Woman alone | 0.79 (0.57-1.09) | 0.81 (0.51-1.27) |
| Woman and Partner | 1.13 (0.92-1.38) | 0.98 (0.70-1.37) |
| **Major purchases** |  |  |
| Partner alone | 1 | 1 |
| Woman alone | 0.99 (0.76-1.30) | 1.27 (0.91-1.79) |
| Woman and Partner | 1.18 (0.96-1.46) | 1.34 (0.99-1.82) |
| **Visiting** |  |  |
| Partner alone | 1 | 1 |
| Woman alone | 1.21 (0.88-1.66) | 1.01 (0.66-1.53) |
| Woman and Partner | 1.14 (0.93-1.40) | 0.86 (0.63-1.18) |
| **Spending husband’s earnings** |  |  |
| Partner alone | 1 |  |
| Woman alone | 0.87 (0.64-1.18) |  |
| Woman and Partner | 1.09 (0.88-1.36) |  |
| **Cofounders** |  |  |
| **Number of household members** |  |  |
| 7 and above | 1 | 1 |
| Less than 7 | **1.39 (1.16-1.66)^‡^** | **1.22 (1.01-1.49)*** |
| **Distance to nearest health facility** |  |  |
| Big problem | 1 | **1** |
| No big problem | **2.20 (1.74-2.77)^‡^** | **1.89 (1.50-2.39)^‡^** |
| **Children overborn** |  |  |
| 5 and above | 1 | 1 |
| 2-4 | **1.22 (1.01-1.48)*** | 0.90 (0.72-1.12) |
| Less than 2 | **1.45 (1.13-1.86)^†^** | 1.04 (0.76-1.42) |
| **Antenatal care attendance** |  |  |
| Less than 8 visits | 1 | 1 |
| 8 visits and above | 0.97 (0.78-1.22) | 0.99 (0.80-1.23) |
| **Visited by fieldworker** |  |  |
| **No** | 1 | 1 |
| **Yes** | **1.39 (1.12-1.74)^†^** | **1.22 (1.01-1.49)*** |

a p-value above 0.25 at bivariable level, *p-value <0.05, † p-value <0.01 ‡ p-value <0.001.
